# *Clonorchis sinensis* infection contributes to hepatocellular carcinoma progression via enhancing angiogenesis

**DOI:** 10.1371/journal.pntd.0012638

**Published:** 2024-11-11

**Authors:** Caibiao Wei, Junxian Chen, Qiuhai Yu, Yuling Qin, Taijun Huang, Fengfei Liu, Xiaolan Pan, Qiumei Lin, Zeli Tang, Min Fang

**Affiliations:** 1 Department of Clinical Laboratory, Guangxi Medical University Cancer Hospital, Nanning, China; 2 Department of Cell Biology and Genetics, School of Basic Medical Sciences, Guangxi Medical University, Nanning, China; 3 Engineering Research Center for Tissue & Organ Injury and Repair Medicine, Guangxi Medical University Cancer Hospital, Nanning, China; Chung-Ang University, REPUBLIC OF KOREA

## Abstract

**Background:**

*Clonorchis sinensis* (*C. sinensis*) infection plays an important role in the progression of hepatocarcinogenesis. However, its specific role in HCC progression remains unclear. This study aimed to investigate whether *C. sinensis* contributes to angiogenesis in HCC.

**Methods:**

A comprehensive clinical analysis was conducted on 947 HCC patients, divided into two groups: *C*. *sinensis* (-) HCC and *C*. *sinensis* (+) HCC. Kaplan–Meier survival curves and log-rank tests were utilized to assess survival outcomes. Microvessel density (MVD) was evaluated through CD34 immunohistochemistry on hepatectomy specimens. A chemistry analyzer and blood analyzer were employed to measure the concentration of circulating angiogenesis-related biomarkers. Quantitative reverse transcription-PCR (qRT-PCR) was used to analyze the expression of angiogenesis-related genes (CD34, Ang1, Ang2, VEGF, PDGF) in HCC tissues.

**Results:**

*C*. *sinensis* infection was associated with poorer outcomes in HCC patients, with significantly shorter overall survival (OS) (*p* = 0.014) and recurrence-free survival (RFS) (*p*<0.001). Notably, *C*. *sinensis* infection led to an upregulation of MVD in HCC tissues (*p* = 0.041). *C*. *sinensis* (+) HCC patients exhibited significantly higher levels of circulating angiogenesis-related biomarkers, including MONO (*p* = 0.004), EOSO (*p* < 0.001), C3 (*p* = 0.001), FIB (*p* = 0.010), PLT (*p* = 0.003), LDH (*p* = 0.004), GLDH (*p* = 0.003), compared to *C*. *sinensis* (-) HCC patients. Moreover, qRT-PCR analysis revealed that most angiogenesis-related genes were overexpressed in patients with *C*. *sinensis* infection.

**Conclusion:**

*C*. *sinensis* infection is closely associated with inflammatory responses and may promote metabolic reprogramming in HCC, thereby enhancing its malignant characteristics.

## Author summary

*Clonorchis sinensis* (*C*. *sinensis*) is a prominent food-borne parasite prevalent in regions such as China, particularly in Guangxi. *C*. *sinensis* is classified as a class I carcinogen by the International Agency for Research on Cancer for cholangiocarcinoma. Recently, *C*. *sinensis* has also been linked to poor prognosis in hepatocellular carcinoma (HCC) patients. However, the mechanisms through which *C*. *sinensis* infection influences HCC progression, particularly through angiogenesis, are not well understood. In this study, we analyzed clinical data from 947 HCC patients, comparing those with and without *C*. *sinensis* infection. Our findings confirmed that *C*. *sinensis* infection was associated with shorter survival outcomes. Furthermore, *C*. *sinensis* (+) HCC patients exhibited significantly higher rates of metastasis and MVI compared to *C*. *sinensis* (-) HCC patients. Notably, *C*. *sinensis* infection led to an upregulation of microvessel density (MVD) in HCC tissues. Additionally, *C*. *sinensis* (+) HCC patients exhibited significantly higher levels of circulating angiogenesis-related biomarkers and overexpression of angiogenesis-related genes. These results suggest that *C*. *sinensis* may promote HCC progression by inducing angiogenesis. Our study sheds new light on the carcinogenic potential of *C*. *sinensis* and highlights the potential of anti-angiogenic therapies as a promising approach to improve outcomes for HCC patients with *C*. *sinensis* infection.

## Introduction

Hepatocellular carcinoma (HCC) accounts for ~90% of all liver cancers and is associated with high morbidity and mortality [1]. Despite advancements in diagnostic techniques and the development of surgical strategies such as hepatectomy, liver transplantation, local ablation therapy, and transcatheter arterial chemoembolization, HCC remains the only major cancer for which death rates have not improved over the last 10 years [2]. The overall survival (OS) of HCC is poor, with a 5-year survival rate of less than 30% and a median survival of 6–10 months [3].

*Clonorchis sinensis* (*C*. *sinensis*) is one of the most severe food-borne parasites, with an estimated 15 million individuals infected worldwide, including approximately 13 million in China [4]. *C*. *sinensis* imposes a significant burden due to various hepatobiliary morbidities, such as cholangitis, liver cirrhosis, and cholangiocarcinoma. A study in China estimated the annual economic burden of clonorchiasis to be several hundred million yuan, primarily due to direct medical costs and productivity loss [5]. Recent clinical studies have revealed that HCC patients with *C*. *sinensis* infection experience poorer prognosis compared to those without *C*. *sinensis* infection [6,7]. The role of *C*. *sinensis* in the development of HCC involves various mechanisms, including promoting the acquisition of cancer stem cell-like characteristics, inhibiting apoptosis in hepatocarcinoma cells and facilitating epithelial-mesenchymal transition (EMT) [7–9]. It is well acknowledged that *C*. *sinensis* can cause liver cirrhosis, which is a substantial risk factor for the development and progression of HCC [10–13]. However, the clinical impact of *C*. *sinensis* infection in HCC patients and the underlying intricate mechanisms remain incompletely elucidated.

Angiogenesis, one of the six biological capabilities of cancer, plays a key role in cancer growth and metastasis [14,15]. HCC is a typical angio-rich tumor characterized by abnormal angiogenesis [16]. Experimental and clinical data indicate that in HCC tumor progression is associated with angiogenesis, and an increase in microvessel density (MVD) is linked to a poor prognosis [17]. Clinical and preclinical trials have tested the benefit of a variety of anti-angiogenic drugs for the treatment of HCC [18]. Many pro-angiogenic and anti-angiogenic factors, including vascular endothelial growth factor (VEGF), fibroblast growth factor (FGF), angiopoietins (Ang), and platelet-derived growth factor (PDGF), are validated to participate in the modulation of tumor angiogenesis [19,20]. However, the alterations in angiogenesis in HCC with *C*. *sinensis* remain unclear.

In this study, we aimed to investigate the role of *C*. *sinensis* infection in HCC progression, specifically focusing on its impact on angiogenesis. Given the known association between *C*. *sinensis* infection and poorer prognosis in HCC patients, we sought to explore whether *C*. *sinensis* contributes to tumor angiogenesis by promoting MVD and upregulating pro-angiogenic factors. Understanding this mechanism could uncover new therapeutic strategies, particularly the potential use of anti-angiogenic agents for treating HCC patients infected with *C*. *sinensis*.

## Patients and methods

### Ethics statement

The study protocol was approved by the Ethics Committees of the Affiliated Cancer Hospital of Guangxi Medical University (LW2024095).

Upon admission, all patients provided written consent for the analysis and publication of their anonymized medical data for research purposes.

### Study population and data collections

**Fig 1:**
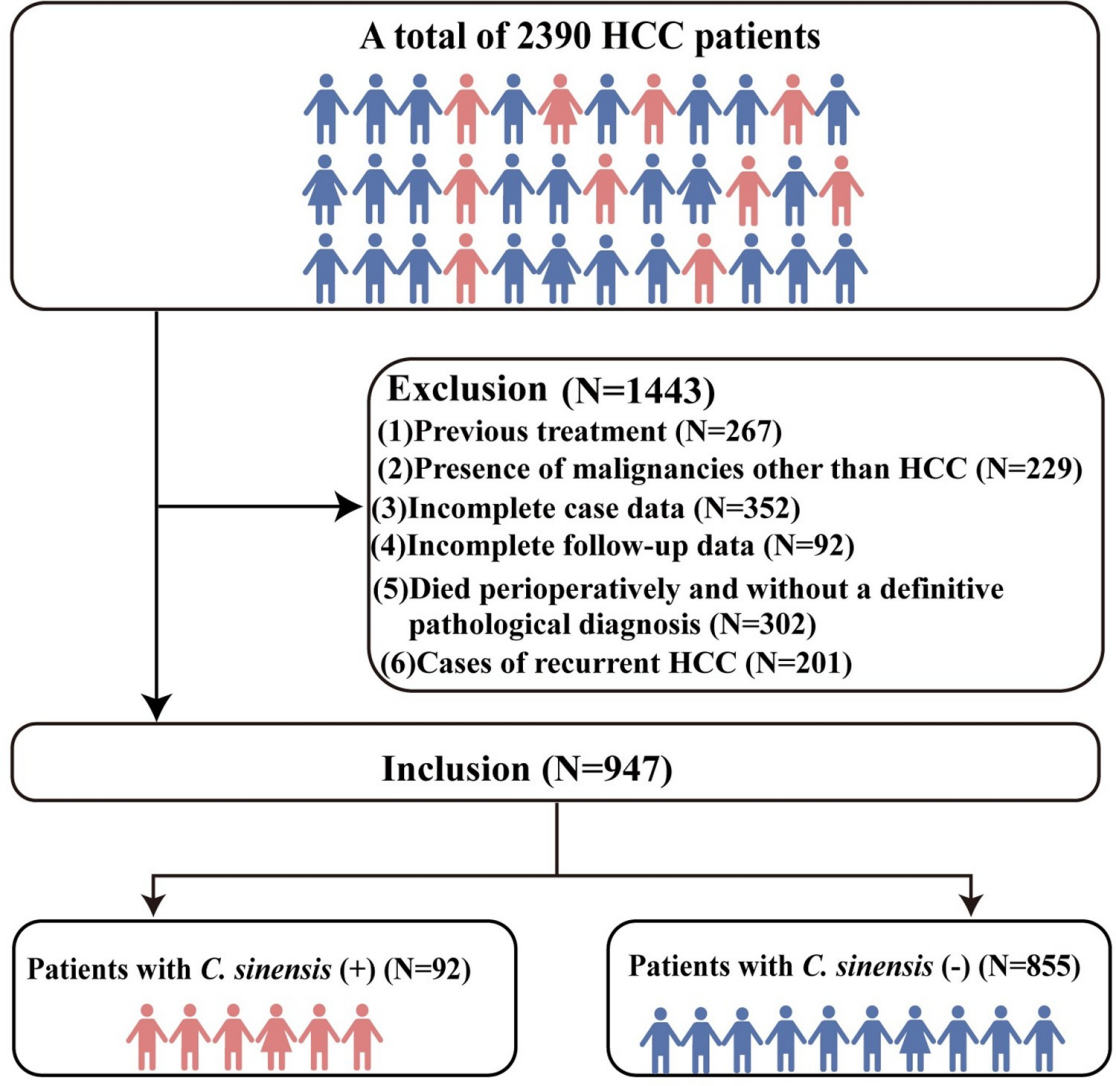
Study flowchart. Data collection was carried out by two independent investigators, CBW and ZLT, with validation conducted by a third investigator, JXC. We obtained a list of HCC patients from the follow-up department and retrieved laboratory data from their initial hospital admissions using the case management system. This data was meticulously recorded in Microsoft Excel. Clinical and laboratory information were collected from the medical records within one week prior to surgery, ensuring accuracy and timeliness. We utilized standardized case report forms to gather demographic details, medical history, treatment regimens, and follow-up outcomes. A retrospective analysis was conducted on patient data incorporated into this study. The data collection included the following: (1) Basic information: gender and age; (2) Clinicopathological information: Barcelona clinic liver cancer (BCLC) stage, tumor size, liver cirrhosis, degree of tumor differentiation (based on the Edmondson-Steiner criteria for tumor histological differentiation) [24], number of tumors, metastasis status and microvascular invasion (MVI) status; (3) Hematological examination: monocyte (MONO), eosinophils (EOSO), circulating complement (C3), fibrinogen (FIB), platelets (PLT), lactate dehydrogenase (LDH), glutamate dehydrogenase (GLDH), and vascular endothelial growth factor (VEGF).

A total of 2390 patients with HCC underwent liver resection at the Guangxi Medical University Affiliated Cancer Hospital between April 2013 and December 2022. The inclusion criteria were as follows: (1) Hepatectomy with a postoperative pathological diagnosis of HCC; (2) Hepatectomy as the first treatment with no history of other malignant tumors; (3) Availability of complete laboratory, pathological, and follow-up information. (4) All patients underwent testing for *C*. *sinensis* infection at the time of initial diagnosis. The following criteria led to the exclusion of 1,443 patients from the study: (1) 267 patients had previous treatments, including radiofrequency ablation or transcatheter arterial chemoembolization before surgery; (2) 229 patients had malignancies other than HCC; (3) 352 patients had incomplete case data; (4) 92 patients lost at follow-up; (5) 302 patients either died perioperatively or had cases without a definitive pathological diagnosis; (6) 201 cases were recurrent HCC. We diagnosed *C*. *sinensis* infection based on the following criteria. Meeting any one of these criteria was sufficient to establish the diagnosis [4,6,21–23]. (1) Preoperative imaging (nuclear magnetic resonance, computed tomography, microscopy, or ultrasound) confirming the presence of *C*. *sinensis* eggs or adult worms on the intrahepatic bile ducts; (2) Preoperative fecal examination showing that the presence of *C*. *sinensis* eggs. (3) Intraoperative or postoperative pathological examination revealing the presence of adult *C*. *sinensis* in the liver or gallbladder. Based on these criteria, a total of 947 patients were included in the analysis, of which 92 patients with *C*. *sinensis* and 855 patients without *C*. *sinensis*. The process for participants in this study is graphically displayed in Fig 1.

### Follow-up routine

Follow-up was conducted by reviewing patient medical records or through medical staff monitoring patients’ recurrence status and the timing of recurrence via telephone. The survival status of each patient was confirmed by death records or by contacting their next of kin (in case of patient demise during follow-up) or a telephone call to the patients themselves. Tumor recurrence was diagnosed based on the imaging manifestations of CT or MRI. Intrahepatic recurrence was diagnosed via imaging, only if the tumor exhibited typical enhancing features. Biopsy confirmed extrahepatic tumors or tumors with atypical imaging features indicative of HCC. All patients underwent follow-up assessments post-surgery, with monthly follow-ups for the first three months. Subsequently, during the first two years, follow-ups occurred every three months. After two years, patients were followed every six months. OS was determined to be the interval between the surgery date and the patient’s HCC-related death or the last follow-up. RFS was determined as the interval between the surgery date and the patient’s tumor recurrence or the date of the last follow-up. The follow-up deadline for this study was August 31, 2023.

### Assessment of circulating angiogenesis-related biomarkers

The levels concentrations of LDH (KWOPK Biotechnology, China), GLDH (ERKN Biotechnology, China) and C3 (Medicalsystem, China) were quantified using a chemistry analyzer (Siemens ADVIA 2400, Germany). Hematological parameters were calculated through an automated blood coulter analyzer (Mindray CAL8000, China). Meanwhile, the concentrations of FIB (Siemens, Germany) was determined by utilizing an automated blood coagulation analyzer (Sysmex-5100, Germany). VEGF (KangHua, China) concentrations were measured by employing a fully automated chemiluminescence immunoassay analyzer (Aurora 1000i, China).

### Assessment of microvessel density by histology and immunohistochemistry

For the evaluation of the angiogenic index, MVD count was used. Microvessel density was evaluated by determining the expression of CD34 through the immunohistochemistry (IHC) method. IHC was performed using the streptavidin-biotin-peroxidase complex method. Histological sections of all paraffin-embedded tissues selected for this study were cut at a thickness of 3–4μm. Liver tissues were fixed in 4% formaldehyde. Sections with a paraffin embedding were deparaffinized. After deparaffinization, slides were hydrated in alcohol, and endogenous peroxidase activity was quenched for 30 min in 3% hydrogen peroxide. Antigen epitope retrieval was induced by microwave heating. To examine the expression pattern of candidate antibodies in HCCs and adjacent tissues, sections were immunostained with primary antibodies overnight at 4°C, using CD34 Polyclonal antibody (dilution 1:100, clone: MX123, Maxim, China). The stained slides were observed and recorded under a bright-field microscope (Olympus BX43, Tokyo, Japan). Two pathologists screened tissue sections at ×40 using an optical microscope (Olympus BX43, Tokyo, Japan) and three areas with the most intense neovascularization were selected. Microvessel counting was performed at × 200 in these areas. MVD positive count < 50 was considered as having low expression, and that ≥ 50 was considered as having high expression.

### Quantitative reverse transcription-PCR (qRT-PCR)

Total RNA was extracted from the HCC tissues using the Trizol reagent (Invitrogen, America). Subsequently, 2μg of RNA was reverse transcribed into cDNA using the Reverse Transcription Master Kit (Takara, Japan) according to the manufacturer’s instructions. qRT-PCR was performed using the qTOWER3 Fluorescence Quantitative PCR Instrument Real-time Fluorescence Quantitative PCR System (Jena, Germany), using TB Green Premix Ex Taq II FAST qPCR (2X) (Takara, Japan). Relative gene expression levels were normalized to the expression of β-actin mRNA and calculated using the 2^-ΔΔCt^ method [25]. The experiments were repeated three times to ensure the consistency and reliability of the results. The primers were designed and synthesized by Synbio Technologies (Shanghai, China). Primer specificity was assessed using the Basic Local Alignment Search Tool available on the National Center for Biotechnology Information website (https://www.ncbi.nlm.nih.gov/). The sequences of the primers for angiogenesis-related genes used in the qRT-PCR assay (S1 Table).

### Statistical analysis

Statistical analysis was conducted using SPSS Version 23.0, R Version 4.2.1 and GraphPad Prism 8.0.2 for Windows. Fisher’s exact or chi-square test was employed to compare group differences in categorical data, expressed as rates. Mann-Whitney U test was used to compare the non-normal continuous data. Further, plotting survival curves and survival analysis were done using the Kaplan-Meier method. The cumulative OS and RFS rates were evaluated using curves and log-rank tests to compare between groups. The independent samples were subjected to a t-test or ANOVA to investigate group differences. Statistical significance was defined if *p*< 0.05.

## Results

### Baseline characteristics

Table 1 provides a detailed overview of the baseline clinical characteristics for all included patients, comparing the two study groups. A total of 947 participants were included in this research, consisting of 847 males (89.44%), with a greater proportion of males in the *C*. *sinensis* (+) HCC group in contrast to the *C*. *sinensis* (-) HCC group (96.74% vs. 88.65%, *p* = 0.017). *C*. *sinensis* (+) HCC patients had a more advanced BCLC stage than *C*. *sinensis* (-) HCC patients (BCLC B-C 59.78% vs. 41.64%, *p<*0.001), higher prevalence of liver cirrhosis (64.13% vs. 51.35%, *p* = 0.020). Furthermore, *C*. *sinensis* (+) HCC patients exhibited significantly higher rates of metastasis (36.96% vs. 22.11%, *p* = 0.001) and MVI (58.70% vs. 47.84%, *p* = 0.048) compared to *C*. *sinensis* (-) HCC patients.

**Table 1:** Patient demographics and clinical characteristics.

Characteristics	Total	*C. sinensis* (-) HCC	*C. sinensis* (+) HCC	
	No. (%)	No. (%)	No. (%)	*P*-value
Total	947	855	92	
Gender				0.017
Female	100 (10.56)	97 (11.35)	3 (3.26)	
Male	847 (89.44)	758 (88.65)	89 (96.74)	
Age(years)				0.357
<58	607 (64.10)	544 (63.63)	63 (68.48)	
≥58	340 (35.90)	311 (36.37)	29 (31.52)	
BCLC stage				<0.001
A	536 (56.60)	499 (58.36)	37 (40.22)	
B~C	411 (43.40)	356 (41.64)	55 (59.78)	
Tumor size				0.531
<5cm	410 (43.29)	373 (43.63)	37 (40.22)	
≥5cm	537 (56.71)	482 (56.37)	55 (59.78)	
Liver Cirrhosis				0.020
Negative	449 (47.41)	416 (48.65)	33 (35.87)	
Positive	498 (52.59)	439 (51.35)	59 (64.13)	
Edmondson grade				0.129
I-II	866 (91.45)	778 (90.99)	88 (95.65)	
III-IV	81 (8.55)	77 (9.01)	4 (4.35)	
Number of tumors				0.678
<2	767 (80.99)	691 (80.82)	76 (82.61)	
≥2	180 (19.01)	164 (19.18)	16 (17.39)	
Metastasis				0.001
Negative	724 (76.45)	666 (77.89)	58 (63.04)	
Positive	223 (23.55)	189 (22.11)	34 (36.96)	
MVI				0.048
Negative	484 (51.11)	446 (52.16)	38 (41.30)	
Positive	463 (48.89)	409 (47.84)	54 (58.70)	

**Abbreviations:** HCC: hepatocellular carcinoma. *C*. *sinensis*: *Clonorchis sinensis*. BLCL: Barcelona clinic liver cancer. MVI: microvascular invasion.

### Prognostic value of *C*. *sinensis* in HCC

**Fig 2:**
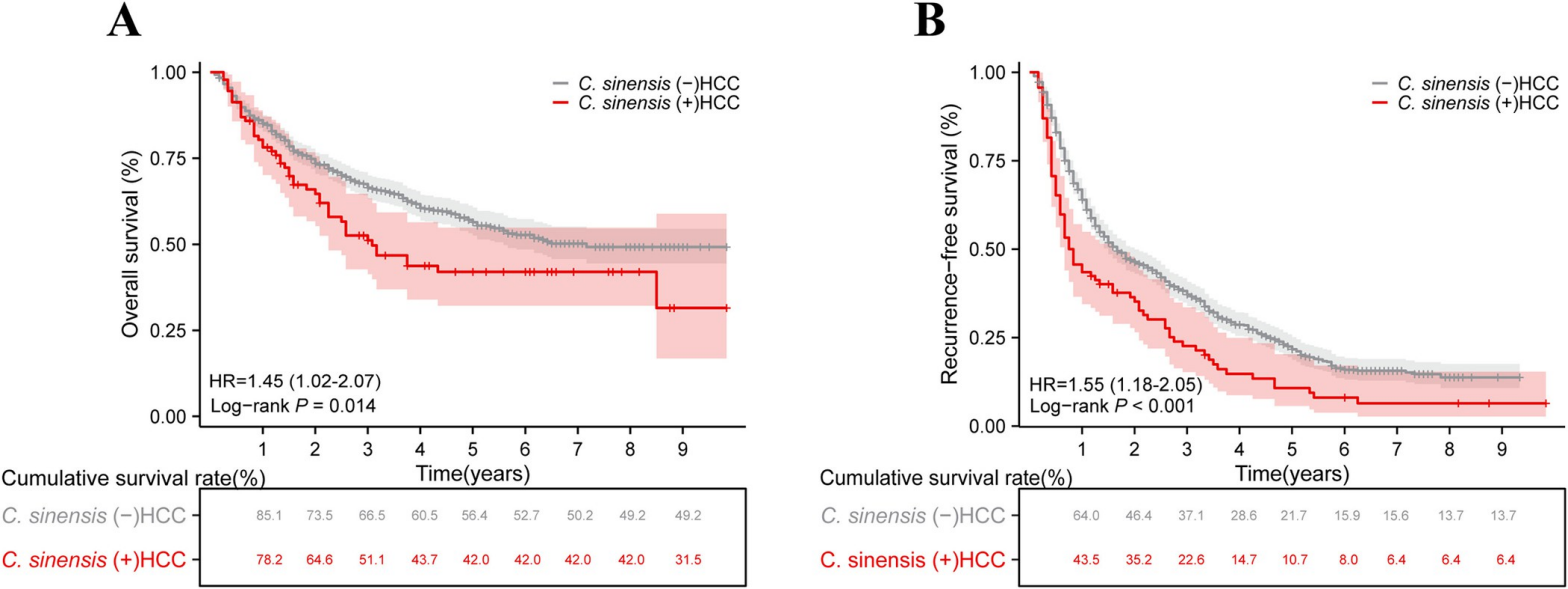
The impact of *C*. *sinensis* on the prognosis of HCC patients after resection. (A) *C*. *sinensis* for Overall survival; (B) *C*. *sinensis* for Recurrence-free survival.

The median OS for the *C*. *sinensis* (+) HCC group was 37 months compared to 86 months for the *C*. *sinensis* (-) HCC group (hazard ratio (HR) = 1.45, 95% confidence interval (CI):1.02 to 2.07; *p* = 0.014) (Fig 2A). The 1-, 3-, and 5-year OS rates for the no *C*. *sinensis* group were 85.1%, 66.5%, and 56.4%, respectively, whereas those for the *C*. *sinensis* group were 78.2%, 51.1%, and 42.0%. The results showed that patients in the *C*. *sinensis* (+) HCC group had worse OS (*p* = 0.014) (Fig 2A). The median RFS was 9.5 months in the *C*. *sinensis* (+) group versus 20 months in the *C*. *sinensis* (-) group (HR = 1.55, 95%CI:1.18 to 2.05; *p*<0.001) (Fig 2B). The 1-, 3-, and 5-year RFS rates for the *C*. *sinensis* (+) group were 43.5%, 22.6%, and 10.7%, respectively, while those for the no *C*. *sinensis* group were 64.0%, 37.1%, and 21.7%. The results showed that patients in the *C*. *sinensis* (+) HCC group had worse RFS (*p*<0.001) (Fig 2B). These findings suggested that *C*. *sinensis* infection is significantly associated with poorer prognosis in HCC patients.

### *C. sinensis* infection enhanced angiogenesis in HCC tissues

**Fig 3:**
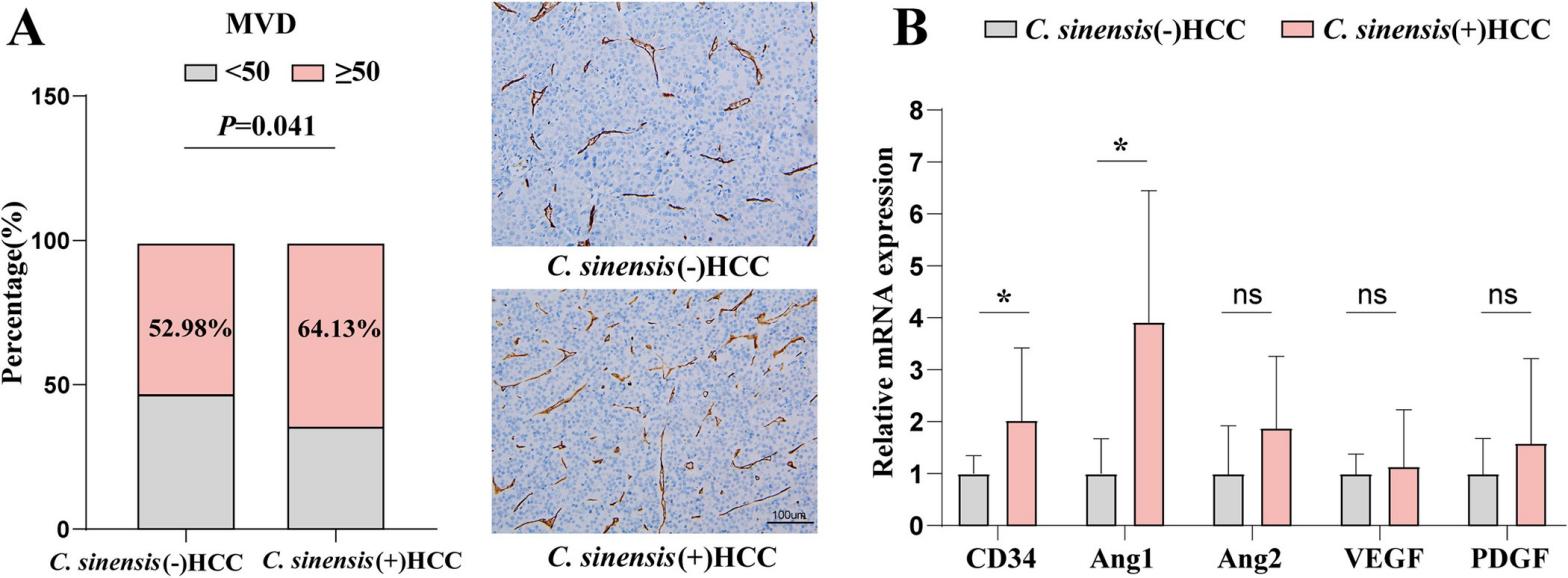
Microvascular density and angiogenesis-related mRNA expressions in the HCC tissues. (A) MVD rates in *C*. *sinensis* (+) (N = 92) and (-) (N = 855) HCC patients. Right pannel: IHC image showing CD34 expression. (B) mRNA level of angiogenesis-related genes in HCC tissues from *C*. *sinensis* (+) and (-) patients (each N = 6). *p < 0.05. Data are presented as means ± SD; n = 6. Student’s t-test was used.

To evaluate the impact of *C*. *sinensis* infection on angiogenesis in HCC patients, we assembled a cohort of 92 *C*. *sinensis* (+) HCC patients and 855 *C*. *sinensis* (-) HCC patients by staining for the vascular marker CD34 in HCC tissues. Our findings revealed that there were more endothelial cells and blood vessels in the *C*. *sinensis* (+) group than in the *C*. *sinensis* (-) group. Particularly noteworthy, MVD rates estimated by CD34 were significantly higher in *C*. *sinensis* (+) HCC patients than in *C*. *sinensis* (-) HCC patients (64.13% vs 52.98%, *p* = 0.041, Fig 3A). We also measured the mRNA expression of genes related to angiogenesis in HCC tissues using qRT-PCR. The *C*. *sinensis* (+) group exhibited higher expression levels of CD34, Ang1, Ang2, VEGF, and PDGF, with only CD34 (*p* = 0.034) and Ang1 (*p* = 0.014) showing statistically significant differences (Fig 3B). These results underscore that *C*. *sinensis* infection promotes angiogenesis, thereby potentially accelerating the progression of HCC.

### *C*. *sinensis* infection accompanied by increased circulating angiogenesis-related biomarkers

**Fig 4:**
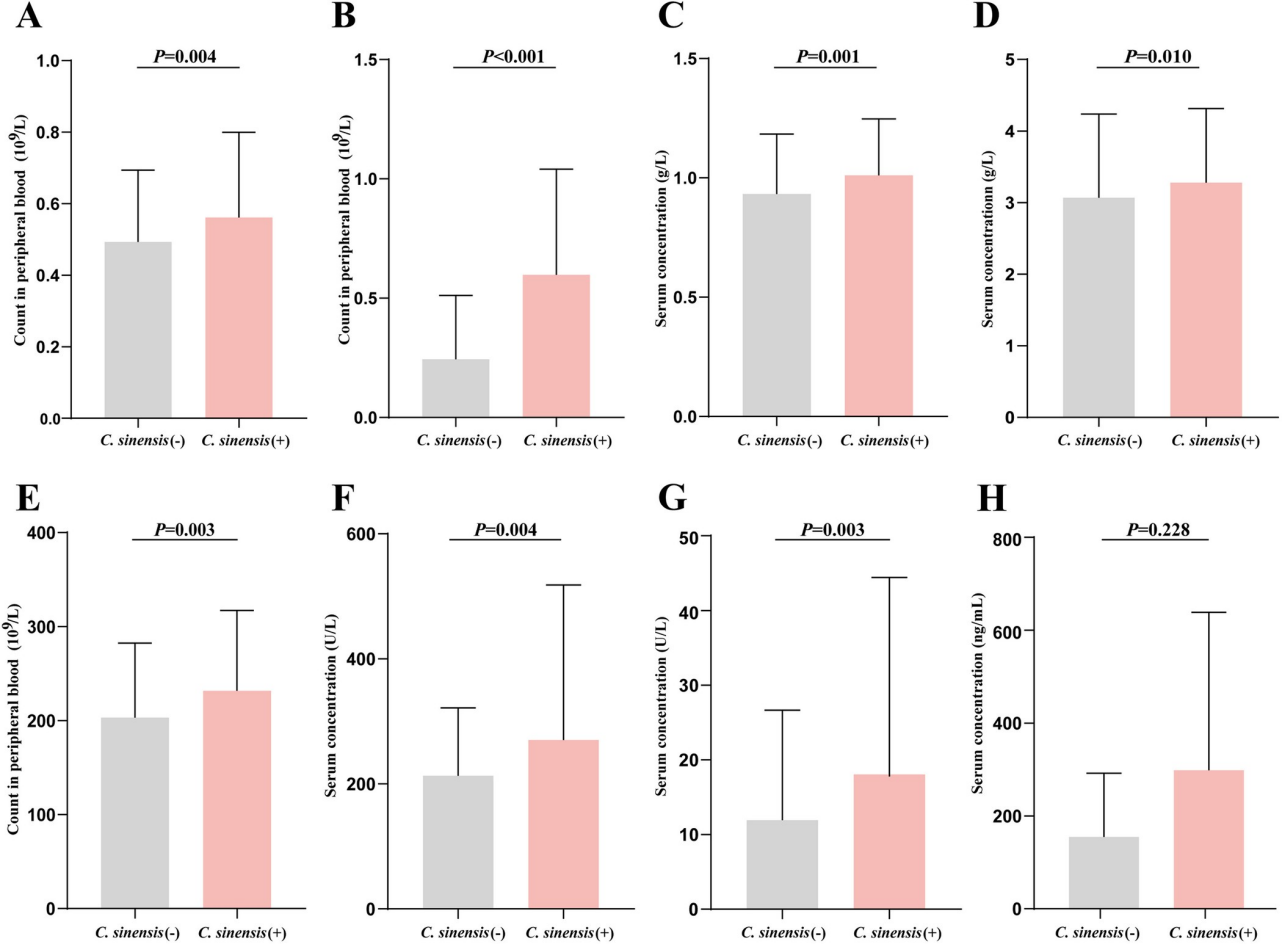
Angiogenesis-related circulating biomarkers in *C*. *sinensis* (+) and (-) HCC patients. (A) Monocyte. (B) Eosinophils. (C) Circulating complement, C3. (D) Fibrinogen. (E) Platelets. (F) Lactate dehydrogenase. (G) Glutamate dehydrogenase. (H) Vascular endothelial growth factor. A-G: data are presented as medians (M) with interquartile ranges (IQR); Mann-Whitney test was used.

Peripheral blood serves as a repository for proangiogenic biomarkers, such as VEGF and PLT, which are pivotal in promoting angiogenesis in diverse physiological and pathological contexts [26]. We measured circulating angiogenesis-related biomarkers in peri-blood samples from HCC patients. The results showed that *C*. *sinensis* (+) HCC patients exhibited higher levels of MONO (*p* = 0.004), EOSO (*p*<0.001), C3 (*p* = 0.001), FIB (*p* = 0.010), PLT (*p* = 0.003), LDH (*p* = 0.004), and GLDH (*p* = 0.003) compared to *C*. *sinensis* (-) HCC patients (Fig 4A–4G). However, there were no statistically significant differences observed in terms of VEGF (Fig 4H). These data suggested *C*. *sinensis* infection might be accompanied by increased circulating angiogenesis-related biomarkers. The overview of how *C*. *sinensis* infection predicts unfavorable prognoses by enhancing circulating angiogenesis-related biomarkers (S1 Fig).

## Discussion

*C*. *sinensis* infection remains a major global health problem and is widely acknowledged as a significant risk factor for HCC development. The carcinogenesis of *C*. *sinensis* encompasses a variety of factors, such as mechanical obstruction, toxic effects of the worms’ ESPs, and immune regulation [12, 27]. In our study, we found that *C*. *sinensis* infection could significantly increase MVD and the expression of proangiogenic biomarkers. This may contribute to *C*. *sinensis*-infected HCC patients exhibiting a shorter OS and RFS. Our findings offer a novel insight into how *C*. *sinensis* promotes HCC progression through an angiogenic mechanism.

Angiogenesis is critical to the growth, invasion, and metastasis of HCC [28]. Our study demonstrated that *C*. *sinensis* infection increases MVD and thus leads to HCC progression. Consistent with our findings, Tingzheng Zhan et al. found that both CD34 gene and protein levels were upregulated in *C*. *sinensis*-infected mice’s liver [13]. Tohada et al. found that decreased CD34 immunostaining indicates a reduction in HCC angiogenesis and metastasis [29]. In agreement with this, we observed that liver tissues from *C*. *sinensis* (+) HCC patients showed higher MVD rates and greater angiogenesis-related mRNA expression than those from *C*. *sinensis* (-) HCC patients, supporting the notion that tumor MVD is an important prognostic factor for HCC, with angiogenesis contributing to HCC progression and poor prognosis. The VEGF signaling pathway has been identified as a major driver of tumor angiogenesis, with VEGF being one of the main vascular endothelial growth factors, indicating its role in the initial phase of angiogenesis [30]. However, we found no difference in the expression of VEGF in either circulating blood or tumor. As the complex process of angiogenesis involves many other factors derived from both tumor cells and endothelial cells. This complexity may explain why VEGF pathway inhibitors often yield temporary improvements followed by resistance rather than enduring clinical responses [31]. Ang1 is a strong agonist of Tie2 activation, while Ang 2 serves as both an antagonist of Ang1-mediated Tie2 phosphorylation and a weak agonist of Tie2 in the absence of Ang1 [32]. Hyung Joon Joo et al. demonstrated that Ang1 significantly enhances the differentiation of CD34 (+) cells into endothelial cells (ECs) [33]. Seiji Noda et al. evaluated the expression of several proangiogenic factors, including CD34 and Ang1, in the context of rheumatoid arthritis, and found increased levels of these markers in synovial cells [34]. With similar findings, we observed that the *C*. *sinensis* (+) group exhibited higher expression of both CD34 and Ang1. This suggests that infection with *C*. *sinensis* may promote the progression of HCC by enhancing angiogenesis.

HCC is a model of inflammation-associated cancer, as inflammation is involved in all stages from tumor initiation to progression and dissemination [35]. Peripheral blood represents a reservoir of inflammatory cells and proteins. Blood monocytes are critical for tumor angiogenesis, as they synthesize inflammatory molecules upon activation [36,37]. More importantly, they express receptors for vascular VEGF and other angiogenic factors, with some serving as circulating precursors of endothelial cells [38,39]. These monocytes can migrate from blood to tumor tissues, where monocyte-derived macrophages infiltrate and locally release a myriad of cytokines, vasoactive agents, matrix metalloproteinases, and growth factors to induce vascular and tissue remodeling or propagate inflammatory responses [36]. Multiple studies have shown that peripheral eosinophils can release angiogenic factors, including VEGF, β-FGF, angiogenin, IL-3, IL-8, and TNF-α, and induce angiogenesis in asthmatic airways and inflammatory tissues [40]. Mounting evidence suggests that complement C3 promotes tumor angiogenesis via C3a-C3aR, which is expressed by monocytes [41,42]. Recent insights emphasize the role of circulating FIB in inflammatory processes and its interaction with various growth factors, encompassing FGF [43,44]. This interaction stimulates endothelial cell proliferation, fostering angiogenesis, and fueling tumor cell growth [45–47]. Platelet has been increasingly recognized as a multipurpose cell influencing a wide range of seemingly unrelated pathophysiologic events. Activation of platelets and release of platelet microparticles leads to the release of a variety of proangiogenic factors, including VEGF, PDGF, FGF, and MMPs [48,49]. We speculate that *C*. *sinensis* infection may be associated with angiogenesis in HCC, potentially through the release of angiogenesis-related factors induced by inflammatory responses. However, further experimental validation is necessary to elucidate the specific mechanisms involved.

Emerging evidence suggests that metabolic reprogramming may affect HCC angiogenesis [50]. Diverse biological and pathological studies have shown that infection with *C*. *sinensis* significantly modifies such as glycometabolism and glutamate metabolism [51]. LDH, the main enzyme involved in glycolysis, is an indirect marker of tumor hypoxia, angiogenesis and poor prognosis in HCC [52,53]. LDH appears to increase HIF-1α and VEGF, which correlates with the angiogenesis pathway [54]. Additionally, GLDH is a key enzyme in glutamine metabolism, which has also been reported to be essential in angiogenesis [55]. We suppose that infection with *C*. *sinensis* promotes angiogenesis by reprogramming the metabolism of HCC.

Nonetheless, our study has a few limitations. First, as a single-center retrospective study, there may have been selection bias. Second, most patients had chronic hepatitis B infection, the results should be validated in other study groups excluding the influence of other aetiologies such as HBV infection, HCV infection and non-viral induced HCC. Furthermore, our study’s sample size is relatively modest, particularly in the female subgroup of HCC patients. The limited number of female patients with *C*. *sinensis* (+) HCC may introduce potential bias. To strengthen the validity and robustness of our findings, it is essential to expand the sample size. Lastly, although our study has illuminated that *C*. *sinensis* associated with HCC anagenesis, the precise underlying mechanism remains not fully explored. Future experiments should be needed to identify the molecular mechanisms through which *C*. *sinensis* infection influences the angiogenesis of HCC.

Collectively, we demonstrated that *C*. *sinensis*-infected HCC patients are associated with shorter survival and more angiogenesis. These findings not only provide insights into the carcinogenic potential of *C*. *sinensis* but also highlight promising strategies to target angiogenesis in *C*. *sinensis*-infected HCCs. Nonetheless, these results should be interpreted with caution and strengthened by large multicenter randomized controlled studies. Additionally, the mechanisms of angiogenesis in this context warrant in-depth investigation.

## Supporting information

S1 Table Sequence of primer sets for qRT-PCR assay. (DOCX)

S1 Fig *C*. *sinensis* infection predicts unfavorable prognoses of HCC through enhancing angiogenesis. (DOCX)

S1 Data Data file. (XLSX)
