# Supplementary material for: Clonorchis sinensis infection contributes to hepatocellular carcinoma progression via enhancing angiogenesis
Source: PLoS Negl Trop Dis. 2024 Nov 11;18(11):e0012638. doi: 10.1371/journal.pntd.0012638 (PMC11554034; doi:10.1371/journal.pntd.0012638)
Supplement: S1 Table — (DOCX) [file pntd.0012638.s001.docx]

**S1 Table. Sequence of primer sets for qRT-PCR assay**

| **Gene** |  | **Primer Sequence** |
| --- | --- | --- |
| **CD34** |  |  |
| Forward primer |  | GACCTTTCAACCACTAGCACTAGC |
| Reverse primer |  | GCCTGAACATTTGATTTCTGCCTTG |
| **Ang1** |  |  |
| Forward primer |  | CCTGATCTTACACGGTGCTGATT |
| Reverse primer |  | GTCCCGCAGTATAGAACATTCCA |
| **Ang2** |  |  |
| Forward primer |  | AACTTTCGGAAGAGCATGGAC |
| Reverse primer |  | CGAGTCATCGTATTCGAGCGG |
| **VEGF** |  |  |
| Forward primer |  | AGGGCAGAATCATCACGAAGT |
| Reverse primer |  | AGGGTCTCGATTGGATGGCA |
| **PDGF** |  |  |
| Forward primer |  | AGTCCAAGGAAGCAATTCACAGTC |
| Reverse primer |  | TTAGGTTGAAGGCTGACTTTAGGTTG |
| **β-actin** |  |  |
| Forward primer |  | CCTGGCACCCAGCACAAT |
| Reverse primer |  | GGGCCGGACTCGTCATAC |
