# Supplementary material for: Clonorchis sinensis infection contributes to hepatocellular carcinoma progression via enhancing angiogenesis
Source: PLoS Negl Trop Dis. 2024 Nov 11;18(11):e0012638. doi: 10.1371/journal.pntd.0012638 (PMC11554034; doi:10.1371/journal.pntd.0012638)

**S1 Fig. *C. sinensis* infection predicts unfavorable prognoses of HCC through enhancing angiogenesis.**


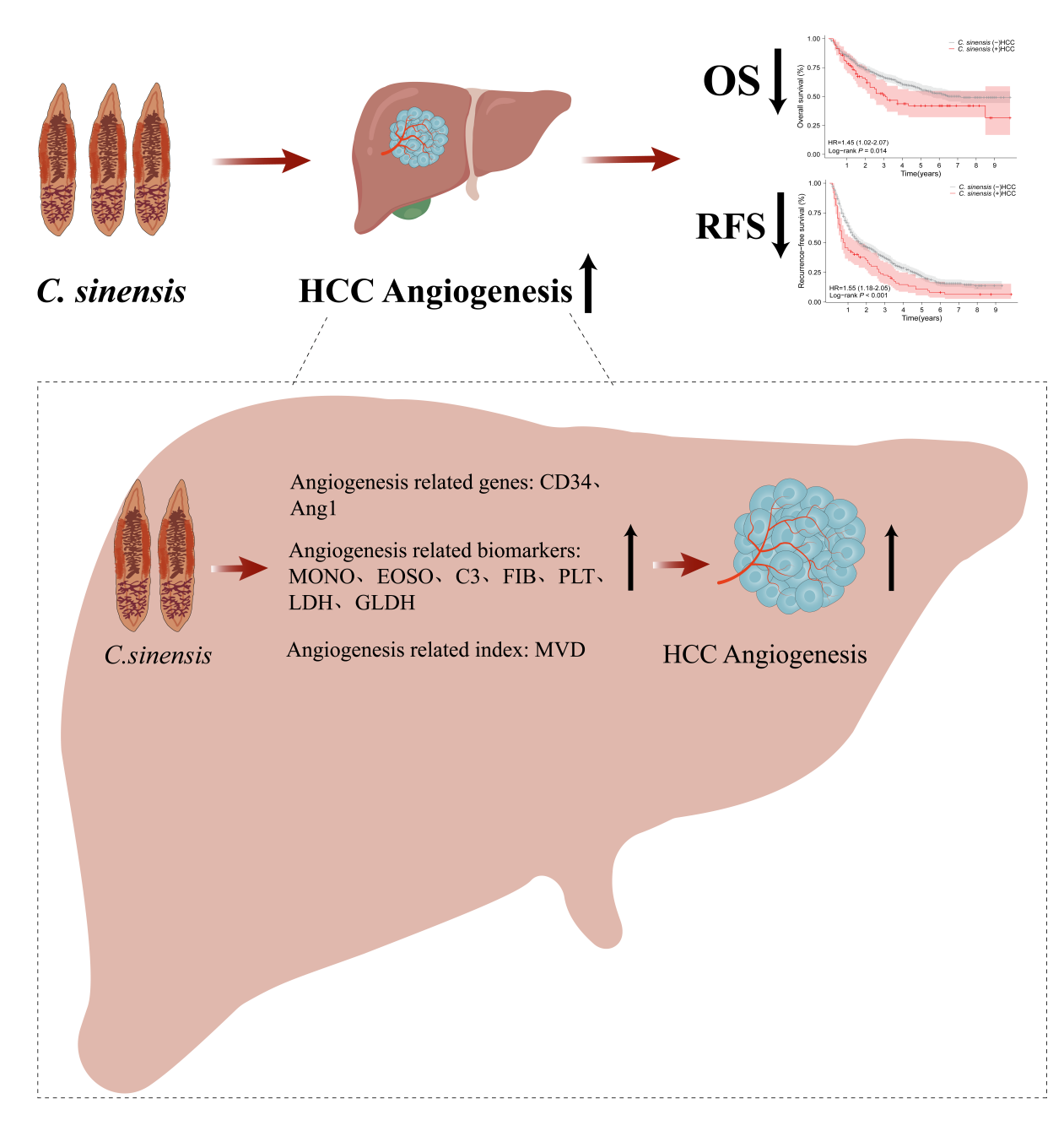

Supplement: S1 Fig — (DOCX) [file pntd.0012638.s002.docx]
